# Pairwise hydrogen addition in the selective semihydrogenation of alkynes on silica-supported Cu catalysts†
†Electronic supplementary information (ESI) available: Additional NMR spectra and data on conversion, NMR signal enhancements and percentages of pairwise hydrogen addition. See DOI: 10.1039/c6sc05276b



**DOI:** 10.1039/c6sc05276b

**Published:** 2016-12-20

**Authors:** Oleg G. Salnikov, Hsueh-Ju Liu, Alexey Fedorov, Dudari B. Burueva, Kirill V. Kovtunov, Christophe Copéret, Igor V. Koptyug

**Affiliations:** a International Tomography Center , SB RAS , 3A Institutskaya St. , 630090 Novosibirsk , Russia . Email: kovtunov@tomo.nsc.ru; b Novosibirsk State University , 2 Pirogova St. , 630090 Novosibirsk , Russia; c Department of Chemistry and Applied Biosciences , ETH Zürich , Vladimir-Prelog-Weg 1-5 , CH-8093 Zürich , Switzerland

## Abstract

Mechanistic insight into the semihydrogenation of 1-butyne and 2-butyne on Cu nanoparticles supported on partially dehydroxylated silica (Cu/SiO_2-700_) was obtained using parahydrogen. Hydrogenation of 1-butyne over Cu/SiO_2-700_ yielded 1-butene with ≥97% selectivity. The surface modification of this catalyst with tricyclohexylphosphine (PCy_3_) increased the selectivity to 1-butene up to nearly 100%, although at the expense of reduced catalytic activity. Similar trends were observed in the hydrogenation of 2-butyne, where Cu/SiO_2-700_ provided a selectivity to 2-butene in the range of 72–100% depending on the reaction conditions, while the catalyst modified with PCy_3_ again demonstrated nearly 100% selectivity. Parahydrogen-induced polarization effects observed in hydrogenation reactions catalyzed by copper-based catalysts demonstrate the viability of pairwise hydrogen addition over these catalysts. Contribution of pairwise hydrogen addition to 1-butyne was estimated to be at least 0.2–0.6% for unmodified Cu/SiO_2-700_ and ≥2.7% for Cu/SiO_2-700_ modified with PCy_3_, highlighting the effect of surface modification with the tricyclohexylphosphine ligand.

## Abstract 2

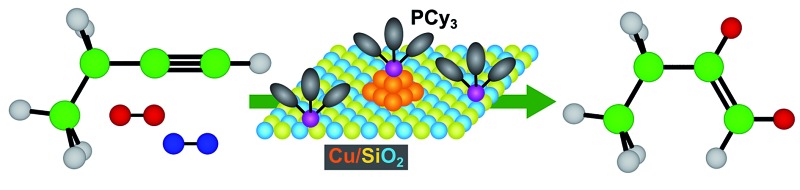
Mechanistic insight into the semihydrogenation of 1-butyne and 2-butyne on Cu nanoparticles supported on partially dehydroxylated silica (Cu/SiO_2-700_) was obtained using parahydrogen.

## Introduction

Semihydrogenation of alkynes is a process used industrially to remove acetylene from ethylene streams and thus avoid poisoning downstream ethylene polymerization catalysts.^1^ It is also an important reaction in organic chemistry applied for the stereoselective synthesis of *Z*-olefins.^2^ Expensive Pd catalysts are currently used for alkyne semihydrogenation, both in industry and in academia (*e.g.*, the Lindlar catalyst).^3^ Research efforts to reduce the required amounts of Pd semihydrogenation catalysts or even totally replace them with cheaper and more sustainable metals identified Cu-based catalysts as a viable alternative.^4–8^ However, further improvement of activity and especially chemo- and stereoselectivity is necessary before Cu can replace the established Pd systems. It has been recently demonstrated by some of us that combining small silica-supported Cu nanoparticles and a phosphorus-based ligand allows one to achieve this goal.^9^ However, a more detailed understanding of the reaction mechanism and the role of the phosphine ligand is critically needed to aid the rational development of improved catalysts.

That said, addressing the molecular mechanisms in heterogeneous catalysis is inherently difficult because of the low abundance of catalytically relevant sites and low sensitivity of many conventional spectroscopic techniques, especially when applied to surfaces.^10^ The latter is a long-standing problem in nuclear magnetic resonance (NMR) and magnetic resonance imaging (MRI).^11^ One strategy to increase the sensitivity utilizes hyperpolarization of nuclear spins, which creates nuclear spin polarization beyond the thermal equilibrium.^12^ Dynamic nuclear polarization (DNP) is a technique that exploits the transfer of polarization from electrons to nuclei.^13,14^ DNP was successfully applied to study surfaces of solid materials,^10,15,16^ including catalysts.^17,18^ Another hyperpolarization technique is parahydrogen-induced polarization (PHIP), which creates non-equilibrium magnetization using a high spin order of parahydrogen (p-H_2_).^19–21^ Parahydrogen itself cannot be detected by NMR because of its zero nuclear spin. However if p-H_2_-derived hydrogen atoms are added to an unsaturated asymmetric substrate as a pair, the corresponding NMR signals of the reaction product are enhanced because the parahydrogen-derived protons retain their spin correlation. This effect was demonstrated with homogeneous^19,22^ and heterogeneous^23–27^ hydrogenation catalysts, the latter of which were utilized both in the liquid^25–27^ and in the gas^23,24^ phases. Applied to heterogeneous catalysis, PHIP provides very valuable mechanistic^28–32^ and kinetic^33^ insights and can be used for MRI visualization of operating catalytic reactors.^34,35^

Here, we use PHIP to obtain mechanistic insight into selective semihydrogenation of alkynes on promising Cu catalysts, in particular to probe whether or not the pairwise hydrogen addition route operates on these systems and how the phosphine ligand might be involved in modifying the reaction pathway and thereby the selectivity of silica-supported Cu nanoparticles.

## Experimental

Cu/SiO_2-700_ and Cy_3_P–Cu/SiO_2-700_ catalysts were prepared using the surface organometallic chemistry approach^36^ grafting [Cu_5_Mes_5_] (Mes = mesityl) onto silica partially dehydroxylated at 700 °C (SiO_2-700_), followed by reduction in H_2_ flow at 300 °C to give Cu/SiO_2-700_. Subsequent impregnation of this material with tricyclohexylphosphine yields the Cy_3_P–Cu/SiO_2-700_ catalyst, as reported in detail elsewhere.^9^

Commercially available 1-butene, 1-butyne (Sigma-Aldrich, ≥99%), 2-butyne (Sigma-Aldrich, 99%) and hydrogen were used as received. For the PHIP experiments, hydrogen gas was enriched with parahydrogen to 50% by passing it through a layer of FeO(OH) maintained at liquid nitrogen temperature (77 K). The 1 : 4 mixtures of 1-butene/p-H_2_ and 1-butyne/p-H_2_ or a 1 : 11 : 5 mixture of 2-butyne/p-H_2_/He were used in the hydrogenation experiments.

Catalysts (20–105 mg) were loaded in the 1/4′′ outer diameter (OD) stainless steel reactor between two pieces of fiberglass tissue in an argon-filled glove box. The reactor was then sealed with two Swagelok valves inside the glove box. All lines between the tank with the gas mixture and the reactor were preliminarily evacuated. The reactor was heated in a tubular furnace; the temperature was varied from 150 to 550 °C. The reaction gas mixture was supplied to the reactor and then to the NMR tube placed inside an NMR spectrometer through a 1/16′′ OD PTFE capillary. All hydrogenation reactions were carried out at atmospheric pressure. The gas flow rate was controlled using an Aalborg rotameter. ^1^H NMR spectra of the reaction mixtures in the gas phase were acquired using a 300 MHz Bruker AV 300 NMR spectrometer using a single π/2 radiofrequency pulse.

## Results and discussion

The Cu/SiO_2-700_ catalyst was prepared using the surface organometallic chemistry approach^36^ by grafting [Cu_5_Mes_5_] onto SiO_2-700_, followed by reduction in H_2_ flow at 300 °C.^9^ This synthetic protocol provided silica-supported Cu(0) nanoparticles with an average size in the range 1.9 ± 0.3 nm, as determined by high-angle annular dark field scanning transmission microscopy after the sample was slowly exposed to air.^9^ Treatment of Cu/SiO_2-700_ with PCy_3_ afforded a surface-modified catalyst (Cy_3_P–Cu/SiO_2-700_) with enhanced selectivity in alkyne batch semihydrogenation.^9^ Both catalysts were then used in the PHIP experiments with 1-butyne, 1-butene or 2-butyne.

**Fig. 1:**
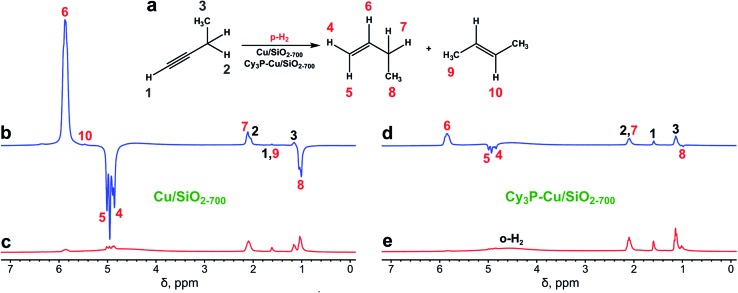
(a) Scheme of 1-butyne hydrogenation. (b and c) ^1^H NMR spectra acquired during 1-butyne hydrogenation with parahydrogen over the Cu/SiO_2-700_ catalyst (b) while the gas was flowing and (c) after rapid interruption of the gas flow and subsequent relaxation of nuclear spins to thermal equilibrium. (d and e) ^1^H NMR spectra acquired during 1-butyne hydrogenation with parahydrogen over the Cy_3_P–Cu/SiO_2-700_ catalyst (d) while the gas was flowing and (e) after rapid interruption of the gas flow and subsequent relaxation of nuclear spins to thermal equilibrium. All spectra were acquired with 8 signal accumulations and are presented on the same vertical scale. The reaction temperature was 450 °C, and gas flow rate was 5.1 mL s^–1^.

The Cu/SiO_2-700_ catalyst showed modest to fair activity in 1-butyne hydrogenation at 350–550 °C, with overall conversions in the 37–81% range, depending on the gas flow rate and temperature (see Table 1), with no significant activity below 300 °C. High selectivity toward 1-butene (≥97%) was always observed (Table 1). Even though hydrogen adsorption on the Cu surface is dissociative,^37,38^ PHIP effects were detected in ^1^H NMR spectra not only for vinylic protons of 1-butene but also for protons of its ethyl moiety as a result of spontaneous transfer of polarization in the Earth's magnetic field (Fig. 1b and c).^39^ The observed signal enhancements (SE) at 7.1 T magnetic field were 25–90 for the CH and 13–60 for the CH_2_ groups of 1-butene, corresponding to 0.2–0.6% and 0.1–0.4% lower boundary estimates for the percentages of pairwise hydrogen addition (*φ*_p_). These values are typical for supported metal catalysts,^24^ and it should be emphasized that the actual contribution of pairwise hydrogen addition is likely higher due to the partial loss of polarization associated with relaxation effects and incomplete adiabaticity of gas transfer into a 7.1 T field.^40^ The difference in the estimated percentages of pairwise addition between CH and CH_2_ groups is explained by the different nuclear spin relaxation rates of these protons.

**Table 1:** Hydrogenation of 1-butyne with parahydrogen over the Cu/SiO_2-700_ catalyst: 1-butyne conversion (*X*), selectivities to different reaction products (*S*), signal enhancements (SE) calculated for vinyl CH and CH_2_ protons of 1-butene, and lower estimates of percentages of pairwise hydrogen addition calculated using SE values

Temperature, °C	Flow rate, mL s^–1^	*X*, %	*S*_1-butene_, %	*S*_2-butene_, %	*S*_butane_, %	SE (CH)	SE (CH_2_)	*φ*_p_ (CH)^*a*^	*φ*_p_ (CH_2_)^*a*^
350	3.8	81	98.5	1.1	0.4	25	13	0.18	0.09
350	5.1	70	98	1.5	0.6	35	29	0.25	0.21
350	6.5	37	100	0	0	88	59	0.63	0.43
450	3.8	71	99	0.8	0.1	42	42	0.31	0.31
450	5.1	62	99	0.6	0.3	67	41	0.48	0.30
450	6.5	55	97	2.0	0.9	79	49	0.57	0.35

^*a*^The losses of polarization caused by relaxation on the way from the reactor to the NMR instrument and the incomplete adiabaticity of this transfer were not taken into account. The difference in the percentages of pairwise addition between the CH and CH_2_ groups is explained by different relaxation rates of these protons.

To assess the stability of the catalyst's performance, it was cooled down to room temperature under H_2_ atmosphere and tested in the hydrogenation of 1-butyne at 350 °C *ca.* 4.5 hours later. The activity of the catalyst decreased dramatically (1-butyne conversion decreased from 81 to 13%), however, a 4–5 fold increase in the SE and *φ*_p_ values was observed (Table S1†), indicating that the catalyst became less active but more selective in terms of pairwise hydrogen addition.

Next, the more selective catalyst,^9^ Cy_3_P–Cu/SiO_2-700_, was tested in the hydrogenation of 1-butyne with parahydrogen (Fig. 1d and e). Similar to Cu/SiO_2-700_, it showed hydrogenation activity only above 250 °C (Table S2†). Cy_3_P–Cu/SiO_2-700_ was significantly less active than Cu/SiO_2-700_ with conversion below 10%. However, it provided higher PHIP enhancements of the 1-butene NMR signals (*ca.* 3–6 fold compared to Cu/SiO_2-700_), and therefore is more prone to pairwise hydrogen addition, assuming that the presence of the ligand does not change the spin relaxation rates significantly. The maximum signal enhancement values were no less than 370 and the maximum contributions of pairwise hydrogen addition were no less than 2.7%, as observed at 350 °C and a 6.5 mL s^–1^ gas flow rate (Fig. S3, Table S2†). Note that as the signals of thermally polarized 1-butene were below the noise level, the SE and *φ*_p_ values were estimated using the signal-to-noise ratio as a reference. As was already mentioned above, losses of polarization due to relaxation and incomplete adiabaticity were not taken into account and therefore the actual SE and *φ*_p_ values can be significantly higher.

In order to gain further insight into the mechanism of the hydrogenation of 1-butyne on these Cu-based catalysts, hydrogenation of 1-butene with parahydrogen was also carried out. The unmodified Cu/SiO_2-700_ catalyst initially showed substantial activity in both the isomerization of 1-butene to 2-butene and hydrogenation to butane even at 150 °C (Table S4†). However, in a short time (∼2–4 min) its activity decreased dramatically (Table S4†). In contrast, the catalyst performance was stable at higher temperatures (250–450 °C). As can be seen from Table S4,† the catalyst was more active in isomerization than in hydrogenation. This activity in the hydrogenation of alkenes to alkanes agrees well with the behavior usually observed in the batch hydrogenation of alkynes, where olefins (primary products) get consumed as soon as there is no more alkyne in the reaction mixture.^9,41^ It is worth noting that no PHIP effects were observed in the hydrogenation of 1-butene on Cu/SiO_2-700_ (Fig. S5†). This observation is in a sharp contrast with the semihydrogenation of 1-butyne under similar reaction conditions and signifies that mechanistic pathways for the hydrogenation of alkynes and alkenes on supported Cu nanoparticles are different.

Similarly, Cy_3_P–Cu/SiO_2-700_ was tested in the hydrogenation of 1-butene. The catalyst did not show any considerable activity in the isomerization or hydrogenation of 1-butene in the 150–550 °C temperature range. This result demonstrates that modification of Cu/SiO_2-700_ with PCy_3_ indeed makes it a very selective catalyst toward hydrogenation of C≡C triple bonds, consistent with the high selectivity to olefins featured by Cy_3_P–Cu/SiO_2-700_ in the liquid phase batch alkyne semihydrogenation.^9^ Note, however, that Cy_3_P–Cu/SiO_2-700_ shows slightly higher activity than Cu/SiO_2-700_ in the semihydrogenation of 1-phenyl-1-propyne at 40 °C in toluene solution,^9^ in contrast with the substantially higher activity of Cu/SiO_2-700_ under the flow conditions utilized in this study. This difference is possibly due to the very different reaction conditions (gas *vs.* liquid phase and temperatures).

Since hydrogenation of terminal and internal alkynes can proceed *via* different mechanisms, Cu/SiO_2-700_ was tested in the hydrogenation of 2-butyne with p-H_2_. Significant activity in the formation of 2-butene and butane was observed starting at 250 °C (see Table S6† for conversions). The catalyst was selective toward 2-butene, but also produced some amounts of butane at 250 and 350 °C. No PHIP effects were observed in 2-butyne hydrogenation over the Cu/SiO_2-700_ catalyst. Similarly, the Cy_3_P–Cu/SiO_2-700_ catalyst showed significant activity above 250 °C. The catalyst was highly selective toward 2-butene (>95%) and in this case, no butane was formed (Table S7†). Then the temperature was increased to 300 °C. At the very first moment at 300 °C and a 6.5 mL s^–1^ gas flow rate, some catalytic activity was observed in the hydrogenation of 2-butyne to 2-butene (Fig. S8,† red trace). Unfortunately, the catalyst rapidly deactivated at the reaction temperature, as evidenced by a rapid decrease in the signals of 2-butene (see Fig. S8,† blue trace) that occurred within minutes. Any PHIP effects, if present, were very small. Interestingly, hydrogenation of 2-butyne over more common heterogeneous hydrogenation catalysts such as Pd/TiO_2_ and Rh/TiO_2_ featured pronounced PHIP effects for 2-butene and butane (Fig. S9†).^30^

**Scheme 1:**
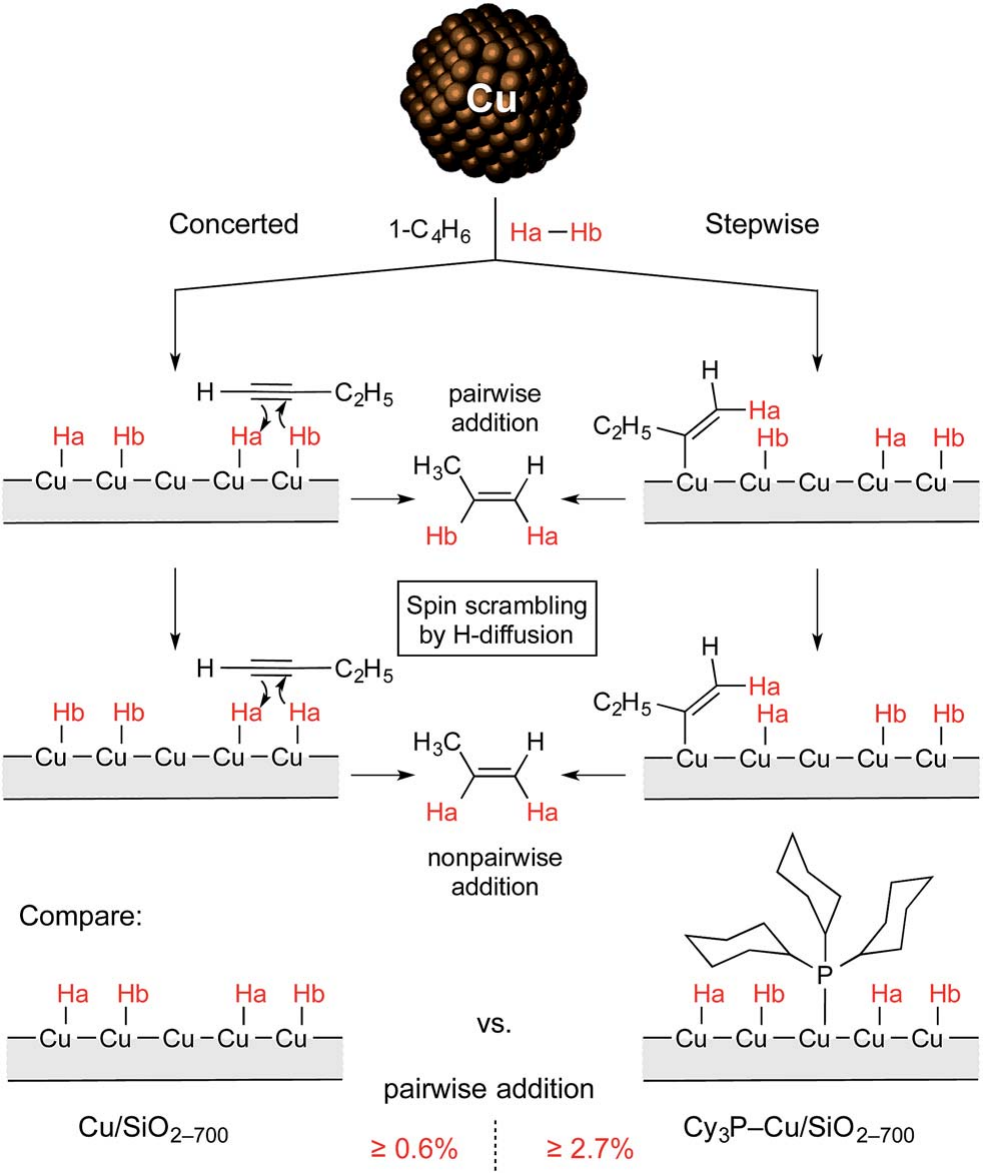
Non-pairwise and pairwise routes of hydrogen addition to 1-butyne over Cu/SiO_2-700_ and Cy_3_P–Cu/SiO_2-700_ catalysts.

Thus, it was found that treatment of the Cu/SiO_2-700_ catalyst with PCy_3_ leads to a significant increase in selectivity toward the formation of alkenes in the flow semihydrogenation of alkynes. Moreover, the modified catalyst provided a higher intensity of PHIP effects pointing to a higher contribution of pairwise hydrogen addition with this material (Scheme 1). These results can tentatively be explained by the selective binding of the PCy_3_ ligand to more active but unselective Cu surface sites, which in turn can impede the migration of hydrogen atoms, making pairwise addition of hydrogen more probable. A significant increase in the chemoselectivity of hydrogenation at the expense of catalytic activity confirms this hypothesis, as such an effect is typically observed with ligand-induced catalyst poisoning in heterogeneous catalysis.

## Conclusions

We reported the first study on selective semihydrogenation of alkynes with silica-supported Cu nanoparticles and parahydrogen. PHIP effects were observed for the hydrogenation of 1-butyne on Cu/SiO_2-700_ and were very minor for the hydrogenation of 1-butene and not observed for 2-butyne hydrogenation, indicating that different mechanistic manifolds operate with these substrates. Modification of the Cu/SiO_2-700_ catalyst with PCy_3_ leads to an increase in the selectivity of 1-butyne hydrogenation to nearly 100% (with by-products below the detection limit of NMR), although it was found to make this catalyst significantly less active. The remarkable selectivity of Cy_3_P–Cu/SiO_2-700_ also manifested in no activity in the hydrogenation of 1-butene, and no formation of butane from 2-butyne. Cy_3_P–Cu/SiO_2-700_ provided no less than 2.7% pairwise hydrogen addition to 1-butyne, with the actual value likely to be higher due to polarization losses *via* relaxation. Our results demonstrate that copper-based catalysts are promising inexpensive alternatives to heterogeneous noble metal catalysts for the semihydrogenation of alkynes in the flow and the production of hyperpolarized gases.
