# Supplementary material for: Pairwise hydrogen addition in the selective semihydrogenation of alkynes on silica-supported Cu catalysts
Source: Chem Sci. 2016 Dec 20;8(3):2426–30. doi: 10.1039/c6sc05276b (PMC5369404; doi:10.1039/c6sc05276b)
Supplement: Supplementary file 1 [file SC-008-C6SC05276B-s001.pdf]

Electronic Supplementary Material (ESI) for Chemical Science.
This journal is © The Royal Society of Chemistry 2016

# Supporting Information for

# Pairwise Hydrogen Addition in the Selective Semihydrogenation of Alkynes on Silica-Supported Cu Catalysts

*Oleg G. Salnikov,*[a,b] *Hsueh-Ju Liu,*[c] *Alexey Fedorov,*[c] *Dudari B. Burueva,*[a,b] *Kirill V. Kovtunov,*[a,b,†] *Christophe Copéret,*[c] *Igor V. Koptyug*[a,b,†]

[a] International Tomography Center, SB RAS, 3A Institutskaya St., 630090 Novosibirsk, Russia

[b] Novosibirsk State University, 2 Pirogova St., 630090 Novosibirsk, Russia

[c] Department of Chemistry and Applied Biosciences, ETH Zürich, Vladimir-Prelog-Weg 1-5, CH-8093 Zürich, Switzerland

**Table S1.** Comparison of $Cu/SiO_{2\text{-}700}$ catalyst performance in 1-butyne hydrogenation with parahydrogen in two tests at 350 °C and 3.8 mL/s gas flow rate. Between these tests the catalyst was held in $H_2$ atmosphere at room temperature for ~ 4.5 hours.

| # of test | $X$, % | $S_{1\text{-butene}}$, % | $S_{2\text{-butene}}$, % | $S_{butane}$, % | SE (CH) | SE ($CH_2$) | $\varphi_p$ (CH) | $\varphi_p$ ($CH_2$) |
|---|---|---|---|---|---|---|---|---|
| 1 | 81 | 98.5 | 0.9 | 0.3 | 25 | 13 | 0.18 | 0.09 |
| 2 | 13 | 100 | 0 | 0 | 120 | 56 | 0.85 | 0.41 |

**Table S2.** The results obtained in 1-butyne hydrogenation with parahydrogen over $Cy_3P\text{-}Cu/SiO_{2\text{-}700}$ catalyst: 1-butyne conversion ($X$), selectivity to 1-butene ($S_{1\text{-butene}}$), signal enhancements (SE) calculated for vinyl CH and $CH_2$ protons of 1-butene, and lower estimates of percentages of pairwise hydrogen addition calculated using SE values.

| Temperature, °C | Flow rate, mL/s | $X$, % | $S_{1\text{-butene}}$, % | SE (CH) | SE ($CH_2$) | $\varphi_p$ (CH)[a] | $\varphi_p$ ($CH_2$)[a] |
|---|---|---|---|---|---|---|---|
| 250 | 6.5 | ≤2.8[b] | 100 | 89[b] | 68[b] | 0.64[b] | 0.49[b] |
| 350 | 3.8 | 6.9 | 100 | 69 | 38 | 0.50 | 0.27 |
| 350 | 5.1 | 4.3 | 100 | 200 | 105 | 1.4 | 0.76 |
| 350 | 6.5 | ≤1.7[b] | 100 | 370[b] | 280[b] | 2.7[b] | 2.0[b] |
| 450 | 3.8 | 11 | 100 | 23 | 12 | 0.17 | 0.09 |
| 450 | 5.1 | 10 | 100 | 33 | 19 | 0.24 | 0.14 |
| 450 | 6.5 | 8.5 | 100 | 61 | 45 | 0.44 | 0.32 |

[a]The difference in percentages of pairwise addition between CH and $CH_2$ groups is explained by different relaxation rates of these protons.

[b]The signals of thermally polarized 1-butene were below the noise level. Therefore, the upper estimates of conversion and lower estimates of SE and percentages of pairwise hydrogen addition were calculated using the signal-to-noise ratio (this assumes that the signals of butane do not exceed the level of noise in the spectra).

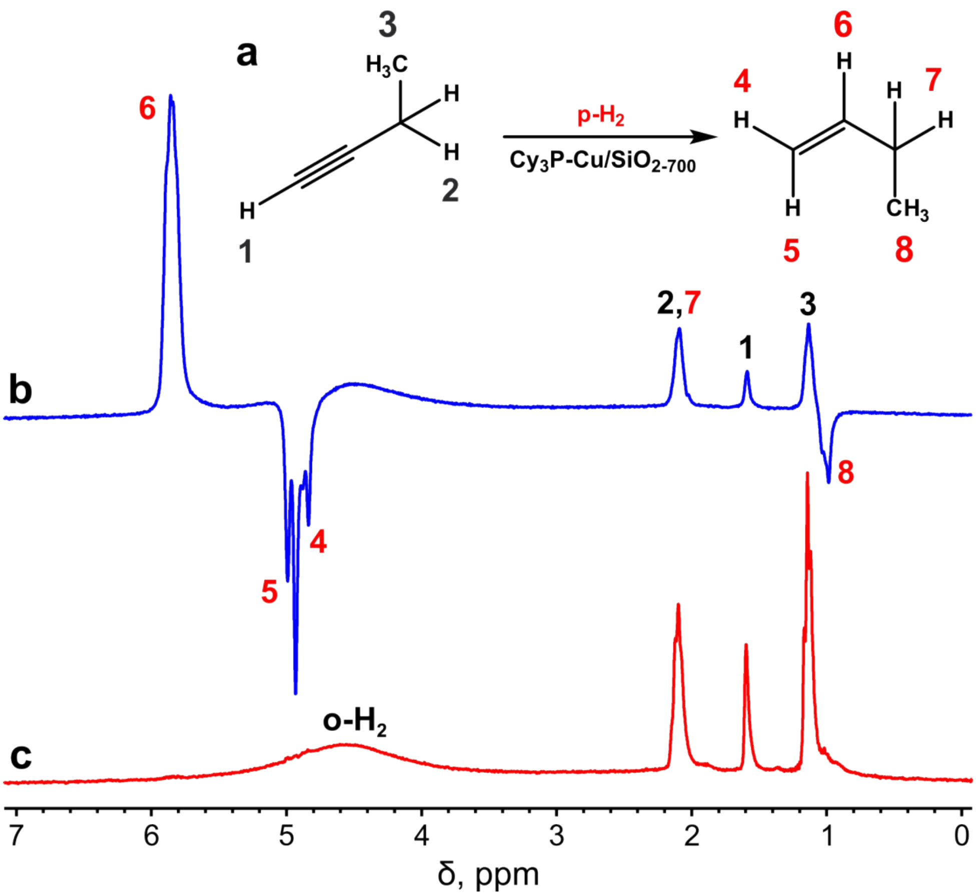


**Figure S3.** (a) Scheme of 1-butyne hydrogenation. (b) $^1$H NMR spectra acquired in 1-butyne hydrogenation with parahydrogen over $Cy_3P$-Cu/$SiO_{2-700}$ catalyst at 350 °C with 6.5 mL/s gas flow rate and (c) after rapid interruption of the gas flow. Both spectra were acquired with 8 signal accumulations.

**Table S4.** The results obtained in 1-butene hydrogenation with parahydrogen over Cu/$SiO_{2-700}$ catalyst: 1-butene conversion (*X*), and selectivities to different reaction products (*S*).

| Temperature, °C | Flow rate, mL/s | *X*, % | $S_{2\text{-butene}}$, % | $S_{\text{butane}}$, % |
|---|---|---|---|---|
| 150 | 1.3 | 78 | 68 | 32 |
| 150 | 2.5 | 80 | 72 | 28 |
| 150 | 3.8 | 7 | 36 | 64 |
| 150 | 2.5[a] | 3 | 11 | 89 |
| 250 | 1.3 | 84 | 74 | 26 |
| 250 | 2.5 | 61 | 69 | 31 |
| 250 | 3.8 | 57 | 69 | 31 |
| 250 | 5.1 | 45 | 57 | 43 |
| 300 | 2.5 | 79 | 77 | 23 |
| 300 | 3.8 | 67 | 72 | 28 |
| 300 | 5.1 | 75 | 68 | 32 |
| 300 | 8.0 | 86 | 56 | 44 |
| 350 | 2.5 | 72 | 73 | 27 |
| 350 | 3.8 | 71 | 67 | 33 |
| 450 | 2.5 | 82 | 67 | 33 |
| 450 | 3.8 | 79 | 64 | 36 |

[a]After partial catalyst deactivation (see the main article).

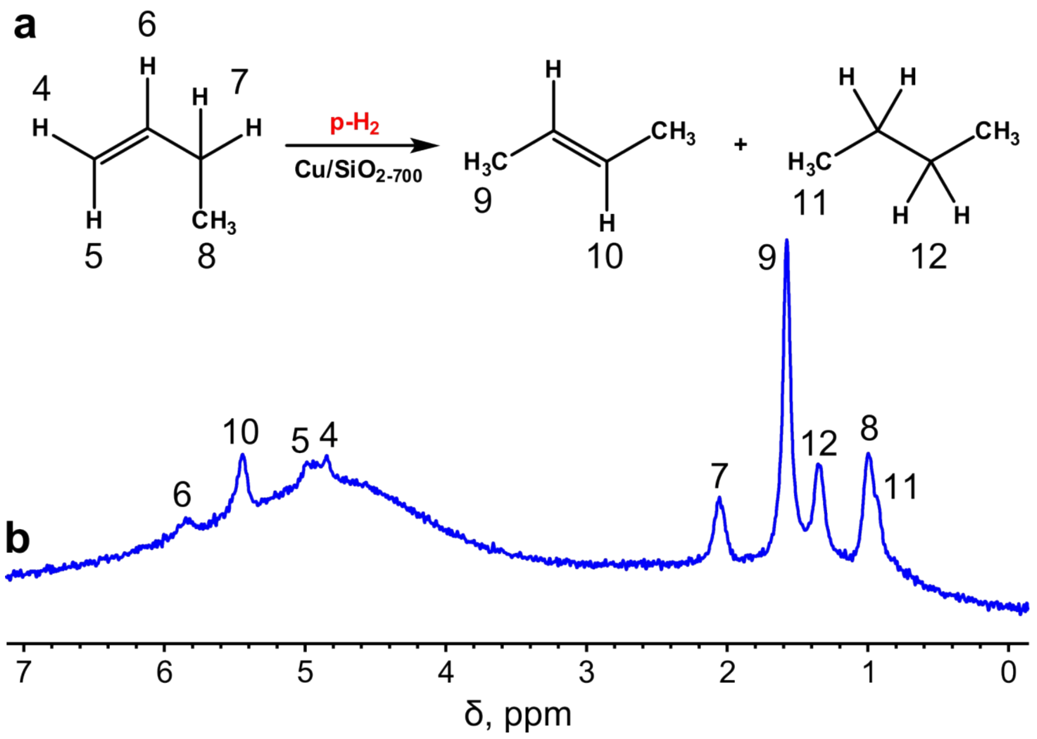


**Figure S5.** (a) The reaction scheme of 1-butene hydrogenation. (b) $^1$H NMR spectrum acquired in 1-butene hydrogenation with parahydrogen over Cu/$SiO_{2\text{-}700}$ catalyst at 300 °C while the gas was flowing at 5.1 mL/s flow rate. The spectrum was acquired with 8 signal accumulations.

**Table S6.** The results obtained in 2-butyne hydrogenation with parahydrogen over Cu/$SiO_{2\text{-}700}$ catalyst: 2-butyne conversion (*X*), and selectivities to different reaction products (*S*).

| Temperature, °C | Flow rate, mL/s | *X*, % | $S_{2\text{-butene}}$, % | $S_{\text{butane}}$, % |
|---|---|---|---|---|
| 250 | 2.5 | 63 | 72 | 28 |
| 250 | 3.8 | 31 | 77 | 23 |
| 350 | 2.5 | 63 | 79 | 21 |

**Table S7.** The results obtained in 2-butyne hydrogenation with parahydrogen over $Cy_3$P-Cu/$SiO_{2\text{-}700}$ catalyst: 2-butyne conversion (*X*), and selectivities to different reaction products (*S*).

| Temperature, °C | Flow rate, mL/s | *X*, % | $S_{2\text{-butene}}$, % | $S_{\text{butane}}$, % |
|---|---|---|---|---|
| 250 | 0.4 | 27 | 100 | 0 |
| 250 | 5.1 | 28 | 100 | 0 |
| 300 | 0.4 | 40[a] | 100 | 0 |

[a]This value was estimated from the NMR spectrum of a flowing gas; partial suppression of NMR signals due to incomplete relaxation of protons to 7.1 T magnetic field was not taken into account. Therefore, the conversion estimated here is less accurate.

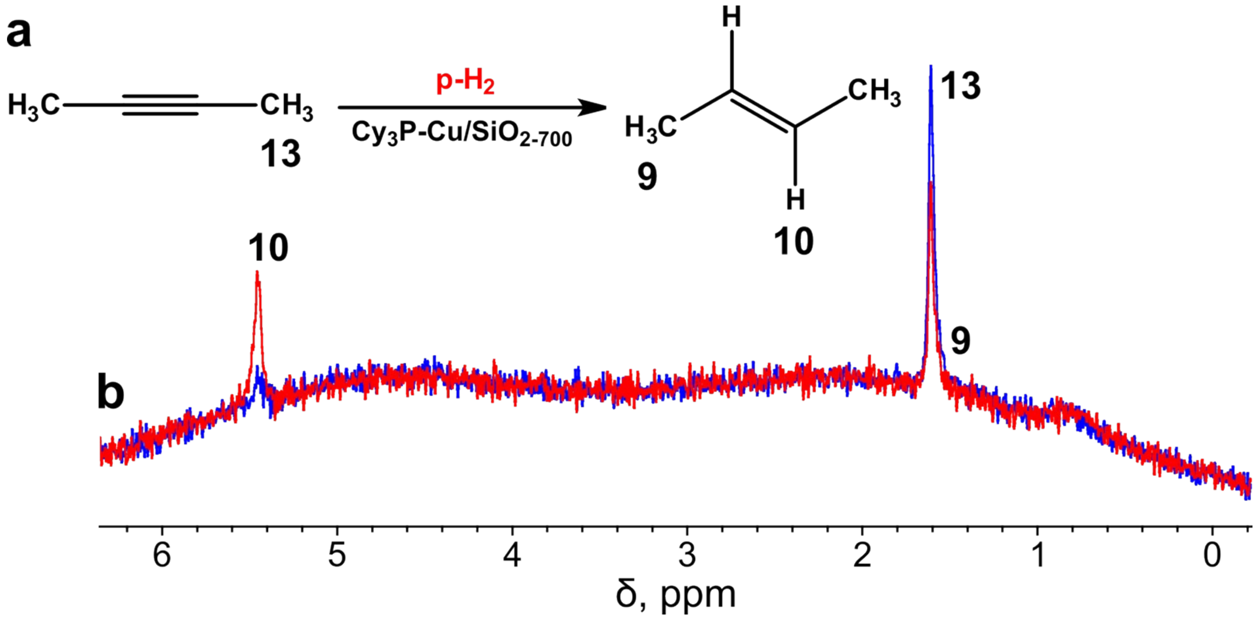


**Figure S8.** (a) Scheme of 2-butyne hydrogenation. (b) $^1$H NMR spectra acquired in 2-butyne hydrogenation with parahydrogen over $Cy_3P$-Cu/$SiO_{2\text{-}700}$ catalyst at 300 °C with flowing gas (6.5 mL/s flow rate). Red trace – spectrum detected right after the temperature was increased to 300 °C, blue trace – spectrum after catalyst deactivation (see main article).

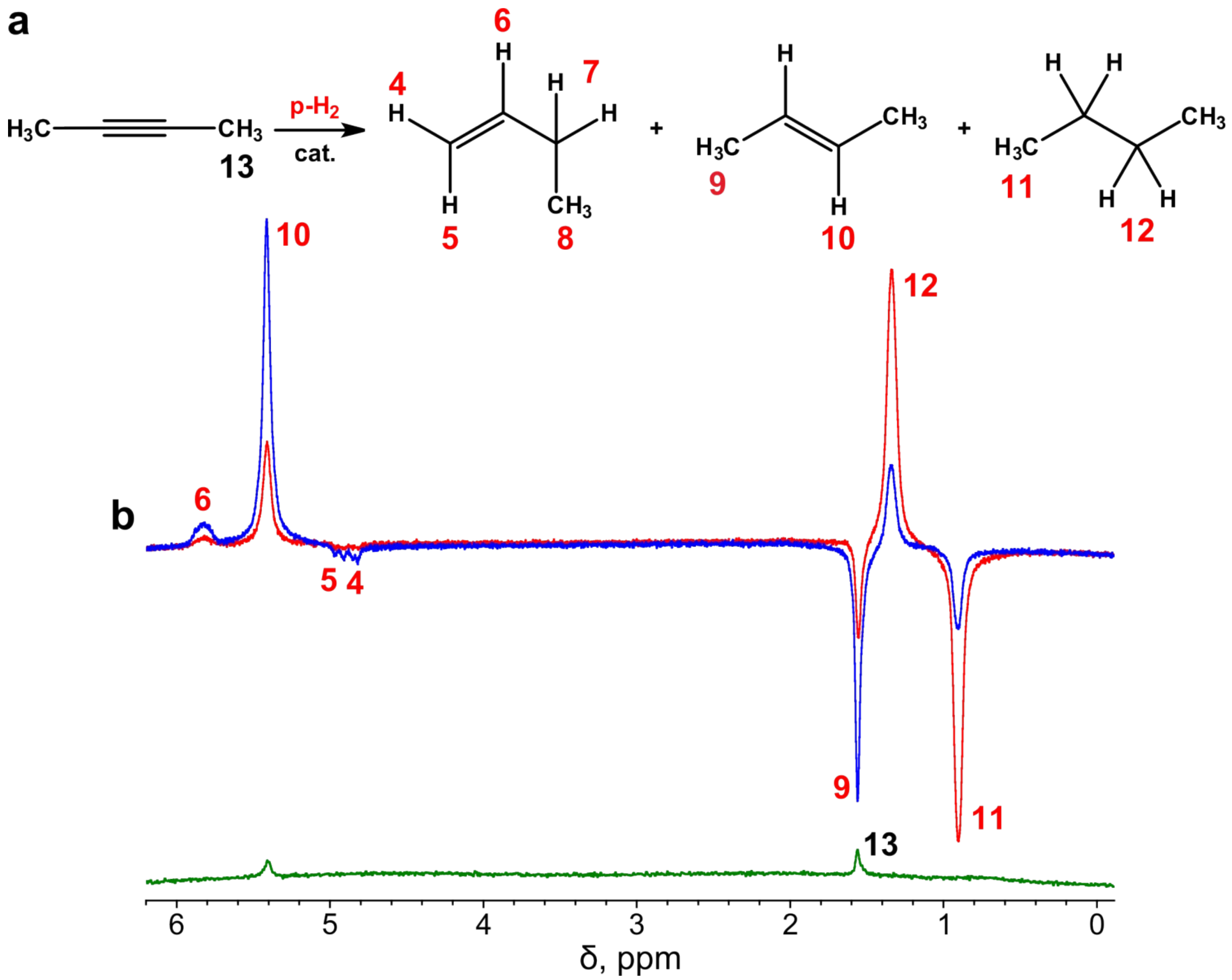


**Figure S9.** (a) The reaction scheme of 2-butyne hydrogenation. (b) $^1$H NMR spectra acquired in 2-butyne hydrogenation with parahydrogen over Pd/$TiO_2$ (red trace), Rh/$TiO_2$ (blue trace) and $Cy_3P$-Cu/$SiO_{2\text{-}700}$ (green trace) catalysts.
